# Structure–Biological Activity Relationships of Extra-Virgin Olive Oil Phenolic Compounds: Health Properties and Bioavailability

**DOI:** 10.3390/antiox9080685

**Published:** 2020-08-01

**Authors:** Paloma Rodríguez-López, Jesús Lozano-Sanchez, Isabel Borrás-Linares, Tatiana Emanuelli, Javier A. Menéndez, Antonio Segura-Carretero

**Affiliations:** 1 Department of Food Science and Nutrition, University of Granada, Campus Universitario s/n, 18071 Granada, Spain; palomarlopez@correo.ugr.es; 2 Research and Development Functional Food Centre (CIDAF), Health Science Technological Park, Avenida del Conocimiento 37, Edificio BioRegión, 18016 Granada, Spain; iborras@cidaf.es (I.B.-L.); ansegura@ugr.es (A.S.-C.); 3 Department of Food Technology and Science, Center of Rural Sciences, Federal University of Santa Maria, Camobi 97105-900, Santa Maria, RS, Brazil; tatiana.emanuelli@ufsm.br; 4 Catalan Institute of Oncology ProCURE (Program Against Cancer Therapeutic Resistance), Ctra. França s/n, Hospital Dr. Josep Trueta de Girona, 17007 Girona, Catalonia, Spain; jmenendez@idibgi.org; 5 Department of Analytical Chemistry, Faculty of Sciences, University of Granada, 18071 Granada, Spain

**Keywords:** extra virgin olive oil phenolic compounds, chemical structure, bioactivity, bioavailability

## Abstract

Extra-virgin olive oil is regarded as functional food since epidemiological studies and multidisciplinary research have reported convincing evidence that its intake affects beneficially one or more target functions in the body, improves health, and reduces the risk of disease. Its health properties have been related to the major and minor fractions of extra-virgin olive oil. Among olive oil chemical composition, the phenolic fraction has received considerable attention due to its bioactivity in different chronic diseases. The bioactivity of the phenolic compounds could be related to different properties such as antioxidant and anti-inflammatory, although the molecular mechanism of these compounds in relation to many diseases could have different cellular targets. The aim of this review is focused on the extra-virgin olive oil phenolic fraction with particular emphasis on (a) biosynthesis, chemical structure, and influence factors on the final extra-virgin olive oil phenolic composition; (b) structure–antioxidant activity relationships and other molecular mechanisms in relation to many diseases; (c) bioavailability and controlled delivery strategies; (d) alternative sources of olive biophenols. To achieve this goal, a comprehensive review was developed, with particular emphasis on in vitro and in vivo assays as well as clinical trials. This report provides an overview of extra-virgin olive oil phenolic compounds as a tool for functional food, nutraceutical, and pharmaceutical applications.

## 1. Introduction

Extra-virgin olive oil (EVOO) represents one of the symbols of the highly valued Mediterranean diet. It is the typical lipidic source in the cuisine of the Mediterranean countries and its consumption has been associated with reduced morbidity and slowing disease progression such as cardiovascular, neurodegenerative, or even cancer diseases [1,2,3,4,5,6,7,8,9,10,11]. On the basis of all of these considerations, EVOO could be considered as a functional food. Although the European regulation does not have a current definition for functional food, it could be defined as follows: “food products can only be considered functional if together with the basic nutritional impact it has beneficial effects on one or more functions of the human organism, thus either improving the general and physical conditions or/and decreasing the risk of diseases evolution” [12,13].

The beneficial impact of EVOO in the human organism is well established, and it is due to its composition. The predominant fatty acid present in the virgin olive oil is monounsaturated oleic acid (68–82% of the total fatty acids in olive oil), which has been widely studied, and its benefits have been established [7,8,10,11]. Moreover, there is a fraction of microconstituents, such as phytosterols, squalene, tocopherols, phenolic compounds, terpenic acid derivatives, etc. Among them, phenolic compounds, which are known for their remarkable antioxidant activity [1,2,14,15], have attracted the attention of researchers belonging to different knowledge areas. They are characterized by a complex mixture of compounds, which occur in the form of simple phenols, lignan derivatives, secoiridoids, and flavonoids. Secoiridoids and alcoholic phenols (mainly hydroxytyrosol) are present in high amounts in virgin and extra-virgin olive oil [2,5]. These phenolic compounds are characterized by a broad spectrum of biological activities, such as reducing the morbidity and slowing the progression of diseases associated with oxidative stress, due to their well established antioxidant activity [16].

Indeed, over the last decades, research into EVOO phenolic compounds has shown the role that these compounds may play in the prevention or slowing down the development of certain pathologies [17,18,19]. For this reason, The European Food Safety Authority (EFSA) has approved in 2011 a health claim stating that the dietary intake of virgin olive oil (poly) phenols is able to protect blood lipids from oxidative damage. The panel considers that in order to bear the claim, 5 mg of hydroxytyrosol and its derivatives should be consumed daily [20,21]. In addition to their widely studied beneficial effects, the latest research has been focused on the metabolic pathways followed by these compounds, their effects on target organs, and even possible delivery strategies as a means of increasing their bioavailability.

The present review studied the relevance of EVOO phenolic compounds with particular emphasis on (a) chemical structure, biosynthesis, and influence factors on the final EVOO phenolic composition; (b) structure–antioxidant activity relationships and other molecular mechanisms in relation to the prevention of many diseases; (c) bioavailability and controlled delivery strategies; (d) alternative source of olive bio-phenols. Thus, the objective of this work is summarizing the scientific state of the art as well as revealing the most innovative arguments for future research based on potential clinical relevance of EVOO phenolic compounds.

## 2. Methods

A literature search was conducted on four electronic databases (PubMed, CrossRef, Scopus and Web of Science). Furthermore, DeCS was used as a descriptor of terms and Mendeley was the reference manager.

### 2.1. Search Strategy

Search terms related to studies of EVOO polyphenols were combined in the following strategies: a) EVOO/Extra virgin olive oil/Olive Oil AND phenol/phenols/polyphenols/phenolic compounds AND properties/health/benefits/oxidative stress/oxidative damage/antioxidant/(anti)cancer/(anti)inflammatory/cardiovascular/digestive disorders/metabolic syndrome; b) EVOO/Extra virgin olive oil/Olive Oil AND phenol/phenols/polyphenols/phenolic compounds AND chemical structure/biosynthesis/bioavailability/absorption/distribution/metabolism/delivery strategies/nanoformulations/sources/synthesis/dopamine.

Keyword searches allowed identifying the relevant literature adequately narrowed down to our research focus using the inclusion criteria described below without limiting the research to specific objectives of this work (e.g., EVOO phenolic compounds and health benefits, biosynthesis and bioavailability).

### 2.2. Inclusion Criteria

Literature review and research articles published since 1 January 2010, with no restriction of language. Due to the high heterogeneity of the proposed objectives, no quality-assessment scale systems were used to evaluate the compiled studies. Manuscript screening was carried out checking the title and abstract or reading the full text. Regarding the possible beneficial heath properties of phenolic compounds, only those trials carried out with phenolic compounds from extra-virgin olive oil and those that were chemically characterized were included. Experimental trials performed in vitro and in vivo (animals and humans) were included and classified according to their potential studied property. Acute and long-term trials were selected to be analyzed in order to compile the described effects over the time-to-event endpoints.

## 3. Results and Discussion

The literature search provided 2976 studies, which were identified before duplicates were discarded. Among these, a total of 116 articles were finally included after applying the inclusion criteria described in Section 2: (a) 29 articles including information about biosynthesis, chemical structure, and influence factors on the final EVOO phenolic composition; (b) 42 articles related to structure–antioxidant activity relationships and other molecular mechanisms in relation to many diseases; (c) 22 of bioavailability and controlled delivery strategies; (d) 15 of alternative source of olive biophenols. Results reveal the most innovative arguments for future research. The most-studied EVOO phenolic compounds were phenolic alcohols and secoiridoids.

### 3.1. Chemical Structure, Biosynthesis, and Influence Factors on EVOO Phenolic Composition

EVOO phenolic compounds have been classified according to their chemical structure into the following main classes: phenolic acids, lignans, flavonoids, phenolic alcohols, secoiridoids, and hydroxy-isocromans [2,5,14,22]. Table 1 shows the chemical structure of the main EVOO phenolic compounds. Phenolic acids identified in EVOO are subdivided into two main groups: hydroxybenzoic acid derivatives, such as *p*-hydroxybenzoic, protocatechuic, vanillic, syringic, and gallic acids; hydroxycinnamic acid derivatives, such as *p*-coumaric, ferulic, caffeic, synaptic, and cinnamic acids [14]. Lignans, which are formed by the condensation of aromatic aldehydes, have also been detected in olive oil samples. (+)-1-Acetoxypinoresinol and (+)-1-pinoresinol were the first characterized and the most concentrated lignans in EVOO [14]. Compounds present in this chemical group are extensively studied because of their high antioxidant capacity through metal-chelating and free radical scavenging activities [2,5,22]. With regard to flavonoids, its structure is formed of two benzene rings joined by a linear three-carbon chain. They can be divided into several groups: flavones, flavonols, flavanones, and flavanols. Two compounds belonging to the first group, apigenin and luteolin, have been the most common ones in EVOO [14].

Phenolic alcohols or phenylethanoids are chemically characterized by the presence of a hydroxyl group attached to an aromatic hydrocarbon group. The main phenolic alcohols described in olive oil are hydroxytyrosol (3,4 dihydroxyphenyl-ethanol or 3,4-DHPEA) and tyrosol (*p*-hydroxyphenyl-ethanol or *p*-HPEA) [14,23].

Secoiridoids come from the secondary metabolism of terpenes. They have a phenyl ethyl alcohol (3,4-DHPEA or *p*-HPEA) linked to elenolic acid or its derivatives and in most cases they are glycosylated. The main glycoside present in the olive fruit is oleuropein, however, during the fruit ripening and the technological process, the aglycone is produced by the β-glucosidase enzyme activity. Therefore, only aglycone derivatives would be present in EVOO. Another phenolic compound of interest belonging to the secoiridoids group is the ligstroside aglycon. Furthermore, derivatives of both oleuropein aglycone and ligstroside aglycone have been detected becoming the major group of phenolic compounds in EVOO [14,24,25,26].

Concerning hydroxy-isocromans, there are only two characterized compounds in EVOO: 1-phenyl-6,7-dihydroxy-isochroman and its derivative 1-(39-methoxy-49-hydroxy) phenyl-6,7-dihydroxy-isochroman [14,27].

Synthesis of EVOO phenolic compounds takes place in the olive fruit through the shikimate pathway, phenylpropanoid metabolism, and mevalonic acid pathway. The latter is responsible for secoiridoid synthesis, and is typical of the *Oleaceae* family, which explains the presence of secoiridoids only in this family of plants [28,29,30]. This synthesis occurs according to the ripening phenomena and in response to interaction with microorganisms. In addition, they are also products of chemical and enzymatic reactions produced by endogenous enzymes such as β-glycosidase, oxidoreductases (polyphenoloxidases), and peroxidases, which hydrolyze phenolic glycosides and oxidase phenolic compounds, respectively. These reactions occur during the technological process of obtaining olive oil [3,14,31]. Consequently, the chemical composition of olive oil phenolics depends on olive fruit ripeness, technological process, variety, and environmental factors [2,5,32,33].

Concerning olive fruit ripeness, some olive-fruit phenolic compounds are not found or are at low concentrations in olive oil. The main phenolic glycosides in the olive fruit tissues are oleuropein and ligstroside [34,35]. However, the activity of hydrolytic and oxidative enzymes over the production steps generate secoiridoid aglycons and derivatives in EVOO, as it has been described above. Indeed, chemical composition is susceptible to change due to chemical and enzymatic reactions that occur during ripening and olive fruit processing [6,14].

The effects of the washing operation, mechanical extraction, malaxation separation systems, storage, and filtration on the individual and total EVOO phenolic compounds have been described in the literature [36]. For instance, oleacein is generated over the oil extraction process, and 3,4-DHPEA and *p*-HPEA concentration is usually low in fresh oils but increases during oil storage due to the hydrolysis of secoiridoids [37,38,39]. Among the other EVOO phenolics, storage did not appear to have a great effect on lignans and flavones [40]. Filtration generates the loss of phenolic compounds, mainly phenolic alcohols and secoiridoids [41]. The best process conditions to produce EVOO with high phenolic content have been described in the literature [42].

With regard to variety, more than 1275 olive cultivars have been identified and classified [33,43]. Depending on the cultivar, the composition of phenolic compounds could show many differences [14,44]. A review by Vossen published in 2013 analyzes the olive phenols content in various countries around the world. Primary world olive tree cultivars organized according to their phenol content include Coratina (Italy), Cornicabra (Spain), Koroneiki (Greece), Moraiolo (Italy), and Picual (Spain) characterized by a very high content of phenolic compounds. They are followed by varieties such a Bosana (Italy), Chemlali (Tunisia), Manzanilla (Spain), Maurino (Italy), Mission (USA) and Picholine (France). Aglandau (France), Ascolano (Italy), Barnea (Israel), Barouni (Italy), Bouteillian (France), Empeltre (Spain), Frantoio (Italy), Hojiblanca (Spain), Kalamon (Grece), Leccino (Italy), and Pendolino (Italy) have a medium content and finally, Arbequina (Spain), Picudo (Spain), Sevillano (Spain), and Taggiasca (Italy) have a low content [43]. It should be taken into account that this study was based on the revision of the published data and differences could be attributed to the different analytical methods used to assess phenolic compounds.

Other external variables, region where olives were grown and its pedoclimatic conditions (soil characteristics, precipitation, temperature, and relative humidity), can modulate phenolic chemical profile. It has been seen that the same variety of olive tree, cultivated under different conditions and location, produce different concentrations of (poly)phenols in EVOO [14,44,45]. For instance, Picual variety cultivated in Andalusia, Catalonia, and Chile presents several differences with regard to the total phenol contents (mg/kg): 664, 609, and 402, respectively [43]. Similar to these results, variation in the phenolic content analyzed in Arbequina EVOO from different regions of Catalonia has also been observed [46]. The major secoiridoid compound quantitated was oleacein, which varied from 78.4 to 116 mg/kg according to the production geographical area.

### 3.2. EVOO Phenolics Structure–Antioxidant Activity Relationships and Other Molecular Mechanisms of these Compounds in Relation to Human Diseases

In recent decades, the effect of EVOO phenolic compounds and their relationship with multiple biological functions have been evaluated in in vitro and in vivo studies. Their impact in the human organism is strongly related to their antioxidant activity [5,47]. They function as an efficient free radical scavenger and metal ion chelator; thus, they counteract the cytotoxic effects of metabolic stress in the organism. The action mechanism could be attributed to the electron donating ability of the hydroxyl groups and subsequent formation of intramolecular hydrogen bonds with free radicals [48,49,50,51,52].

In addition to antioxidant properties, EVOO phenolic compounds have shown anti-inflammatory effects. In this regard, it is important to remark that in vitro studies evinced that hydroxytyrosol derivatives such a hydroxytyrosol acetate and 3,4-dihydroxyphenylglycol showed a special role in the anti-inflammatory effect attributed to EVOO phenolics [53,54]. They have a strong reactive oxygen species (ROS)-scavenging activity, reducing nitrite levels, and downregulated cyclooxygenase-2 (COX-2) expression and prevented the degradation of factors involved in cellular responses to oxidative stress in mammalian cells [54]. These effects add to the already known potent anti-inflammatory properties of EVOO secoiridoids, mainly oleocanthal and oleacein, that is also underlined by the inhibition of COX-2 activity [31]. Nikou et al. in 2019 also found that oleocanthal and oleacein activated cytoprotective pathways promoting healthy aging in both mammalian cells (in vitro) and in a *Drosophila* in vivo model [19]. Such effects included the upregulation of proteasome expression and the suppression of oxidative stress that were likely triggered by nuclear factor erythroid 2-related factor 2 (NFR2) activation, a factor which is related to inflammation [19].

The antioxidant and anti-inflammatory EVOO phenolic compounds synergistic effects have been associated to the bioactivity of EVOO against chronic diseases since the oxidative stress pathways and inflammation are related to different pathologies: i.e., neurodegenerative, digestive disorders, cancer, and metabolic syndrome. As far as neurodegenerative diseases are concerned, hydroxytyrosol is currently the most actively investigated natural polyphenol. Its antioxidant activity influences other systems, for instance, against oxidative damage in vitro in retinal pigment epithelial cells, which occurs in age-related macular degeneration lesions [55]. Moreover, an in vivo assay revealed that oral supplementation of EVOO and specifically hydroxytyrosol reduces brain lipid peroxidation (LPO) and blocks GSH depletion in rats, acting as a powerful brain antioxidant [56]. These effects have been observed in a rat model of brain oxidative damage induced by 3-nitropropionic acid that mimics the neurodegenerative Huntington’s disease.

With regards to digestive disorders, in vitro treatment with phenolic extracts of olive oil counteracts the oxidative and inflammatory effects of oxidized lipids such as hydroperoxides and oxysterols on enterocyte-like Caco-2 cells [57]. In this model, olive oil phenolics have been shown to attenuate the mitogen-activated protein kinase (MAPK)/nuclear factor kappa B pathway, which has been implicated in the pathogenesis of inflammatory bowel diseases [24,57]. These results pointed out the potential protective effect of olive oil phenolics preventing the production of oxidative compounds, modulating pro-inflammatory mediators, or inhibiting the toxic effect of dietary oxidants like oxidized products of cholesterol present in cholesterol-containing foodstuffs [57]. The effect of EVOO phenolic compounds against inflammatory bowel diseases has also been evaluated in in vivo studies. Although no protective effect against colonic inflammation was found in vivo in transgenic HLAB-27 rats [58], other in vivo assays in mice demonstrated an anti-inflammatory effect that prevents digestive disorders including inflammatory bowel disease and acute ulcerative colitis by EVOO polyphenols rich diet [59].

Complementary studies have pointed out the anti-inflammatory capacity of hydroxytyrosol acetate and its importance on acute ulcerative colitis. This compound might provide the beginning of the development of a new strategy for the prevention of ulcerative colitis [60]. Diets enriched with EVOO reduced about 50% the mortality caused by dextran sulphate sodium (DSS) in mice, which induces colonic inflammation, similarly to ulcerative colitis. In addition to these results, hydroxytyrosol supplementation may improve chronic colitis through nitric oxide synthases regulation plus antioxidant capacity [61]. In addition, patients with inflammatory bowel disease are at increased risk for developing colorectal cancer. The impact of diet enriched in polyphenols was evaluated in in vivo models and the results showed less incidence and multiplicity of tumors [59]. 

Concerning cancer diseases and EVOO phenolic compounds, in vitro studies have reported that some phenolic compounds isolated from olive oil have anticancer activity against different types of cancer. Although the molecular mechanism for the anticancer properties of EVOO phenolic compounds could have different cellular targets, it has been described that they can inhibit oncogenic factors, including mutations, catalytic activities of predicted metabolic, and epigenetic targets and interactions that affect DNA methylation [62,63,64,65,66]. Specifically, the inhibition of prostate cancer by a hydroxytyrosol-rich extract from olive mill wastewater was found to be mediated by inhibition of cell proliferation, adhesion, migration, and invasion [17]. In addition to hydroxytyrosol, oleuropein also demonstrated a chemopreventive role in the proliferation of breast cancer cells by inhibiting estrogen-dependent signals [48]. Oleocanthal and oleacein reduced the viability and migration of non-melanoma skin cancer cells, however, tyrosol and hydroxytyrosol showed no effect in this cancer type [18]. On the other hand, metabolites produced by the degradation of EVOO phenolic compounds by gut microbiota may have a chemopreventive effect in colorectal cancer, which is the second most common cancer-related death worldwide [67]. Regarding in vivo studies, one of them has shown a powerful relationship between secoiridoid oleacein and the suppression of functional traits of breast cancer [68].

EVOO phenolic compounds are even related to the prevention or inhibition of metabolic syndrome diseases. An in vitro assay demonstrated that oleacein acts as an inhibitor of lysine-specific histone demethylase 1A (LSD1) a central epigenetic regulator of metabolic reprogramming in diseases associated with obesity, neurological disorders, and cancer [65]. Inhibitory effects were also found against enzymes related to hyperglycemia associated with hypertension, such as α-glucosidase, α-amylase, and angiotensin-converting enzyme (ACE) [69].

Furthermore, reactive oxygen species are critically involved in the endothelial dysfunction contributing to atherosclerosis development. In vitro studies show that EVOO polyphenols are able to lower oxidative stress and inflammatory-related sequelae associated with chronic degenerative diseases [70]. This is due to its ability to modulate genes involved in oxidative tissue damage through the activation of the nuclear erythroid 2-related factor 2 (NRF-2)/antioxidant response element (ARE) and the AMP-activated protein kinase (AMPK) pathways [19,71]. Zrelli et al. studied specifically the hydroxytyrosol function and indicated that it regulates the intracellular reactive oxygen species levels in vascular endothelial cells and provides a molecular basis for the prevention of cardiovascular diseases [72].

Regarding cardiovascular diseases and clinical trials, the present review evaluated studies that relate the consumption of characterized olive oil, rich or enriched in phenolic compounds with cardiovascular prevention factors. Acute [73,74,75] and sustained [75,76,77] trials have been found and they were performed in both healthy [73,74,75,77] and hypercholesterolemic subjects [75,76] or with metabolic syndrome [73]. It can be described that EVOO phenolic compounds intake showed favorable results in modulation of oxidative balance markers of cardiovascular disease [73,74,75]. The results were better in healthy patients [75] and benefits were also observed in insulin sensitivity, glycaemia, modulation of transcription of genes involved in lipid and glucose metabolism, inflammation, and cancer [73], and a significant reduction of oxidized LDL, malondialdehyde, triglycerides, and visceral adiposity index [74]. Another clinical trial on Mediterranean diet and olive oil intake suggests that part of the beneficial effects in reducing oxidative stress and regulation of pro-atherogenic genes are due to the EVOO phenolic compounds. It also suggests the existence of a close relationship between nutrigenomic effects and the decreased risk of cardiovascular disease [77]. Similar results were found in another study conducted in hypercholesterolemic patients, increasing HDL cholesterol levels and improving cardiovascular protection [76].

Although all trials found positive results, it is mandatory to remark that not all trials performed pre-intervention washout periods and only a few combined the intervention with a low phenolic compound diet. Furthermore, both doses supplied (25–50 mL) and phenolic compounds composition, were different. More studies are necessary before definitive conclusions.

Besides the beneficial effects attributed to the EVOO phenolic compounds antioxidant activity, the evidence suggests that EVOO benefits are partially attributable to changes in gene expression [68,78,79]. Thus, a new research area is opened on the possible effects on genetic modulation.

While there are considerable data suggesting benefits of polyphenol intake, it is also essential to consider and study if there is a possibility of toxicity in the consumption of these compounds. Hydroxytyrosol, which is one of the most studied compounds and to which a great antioxidant capacity is attributed, has not reported toxicity and no adverse effects (NOAEL) have been observed up to 500 mg/kg/day (NOAEL) [80]. Phenolic compounds play a key role in the beneficial effects of EVOO on human health and could act as supplement in the pharmaceutical and nutraceutical industry for the treatment and prevention of oxidative stress such as inflammatory and cardiovascular diseases and cancer [81,82,83].

### 3.3. EVOO Phenolics Bioavailability and Controlled Delivery Strategies

The promising beneficial effects of bioactive compounds initially depends on whether the active compound and its concentration at in vitro or in vivo assay become available at the site of action in the human body [23]. Scientific literature supports the idea that most of the dietary phenolic compounds are stable under gastric conditions and reach the intestine, where they can be directly absorbed, metabolized, and distributed to target tissues or continue its biotransformation by colonic bacteria [84,85,86]. For this reason, the main concerns about the evaluation of the effect of EVOO phenols involve bioaccessibility and bioavailability based on absorption and colonic fermentation, distribution, and metabolism.

The absorption mechanism of phenolic compounds is still unclear. In the gastrointestinal tract, olive oil produces a micellar solution. Most EVOO phenolic compounds pass through the mouth and stomach to reach the small intestine and colon without any modification [31]. Hydroxytyrosol and tyrosol have been demonstrated to be the best absorbed phenolics in the intestinal tract (absorption rate ≈ 40–95%) in a dose-dependent mode [3]. Regarding secoiridoids, they remain highly stable in the mouth but suffer significant losses in the gastric, duodenal, and colonic regions, with a recovery rate at the duodenal level ranging between 7% and 34%. Glycosylation and cleavage of glycosidic linkages take part in the secoiridoids absorption, and it is thought that some of them, such as oleacein, are absorbed in the small intestine by passive diffusion through the membrane of intestinal cells [31].

It is also important to consider different factors that can affect the EVOO phenolic compounds absorption such as food matrix. In this concern, better absorption has been found when these compounds were administrated in oil matrix, as a natural component of olive oil. Worst responses were observed with other matrices such as water, yogurt, or even adding it to refined olive oil [87]. These results are supported by the information reported in the literature in relation to nutraceutical formulations. Indeed, a better response was obtained when the phenolic compounds extract was administrated as liquid rather than in capsule form [84]. In this clinical trial based on the bioavailability of phenols from an olive leaf extract, hydroxytyrosol metabolites and low levels of oleuropein were detected in plasma after intake (23–93 min). Although free hydroxytyrosol has been detected in plasma after dietary intake, most absorbed hydroxytyrosol is bioavailable as conjugated metabolites [84,87]. These findings highlight the need for in vitro studies addressing the effects of biologically relevant compounds, such as hydroxytyrosol metabolites, which were the major phenols found in plasma after dietary intake of olive phenols. On the other hand, it seems to be a relationship between bioavailability and gender. Males tend to have higher peak hydroxytyrosol concentrations than females, possibly resulting from differences in human enzymatic activity, however more research is needed before reaching conclusions [88].

Those phenolic compounds that cannot be absorbed in the small intestine will reach the colon, where they can be fermented by gut microbiota [88,89]. The products of colonic fermentation of phenolics can also have beneficial health effects either in situ by promoting intestinal homeostasis and exerting a prebiotic-like effect, or systemically after absorption [84,85,86]. Indeed, EVOO intake has reported benefits in mice gut microbiota compared to other fats and to refined olive oil, through modulating the growth of undesirable bacteria [88]. In addition to this information, data from cell culture assays reveals that EVOO phenols modulate enterocytes response to oxidative and inflammatory stimuli counteracting the pro-oxidant action of oxidized lipid, tert-butyl hydroperoxide (TBH), or a mixture of oxysterols of dietary origin [24,90]. Therefore, both the non-absorbable fraction of phenols and the absorbable phenols before absorption from the intestinal lumen may protect the intestinal mucosa, which is constantly exposed to harmful substances, introduced partly through the diet as unsaturated fatty acids or oxidized cholesterol products, which are mainly responsible for the presence of oxidized species at the colon level [24,90,91,92].

Once absorbed, phenolic compounds are distributed and metabolized throughout the body. A trial conducted in rats reported that hydroxytyrosol accumulates in a dose-dependent manner, in plasma and urine and even accumulates in liver, kidneys, and brain [93]. Furthermore, it seems that hydroxytyrosol can cross the blood–brain barrier and exert a neuroprotective effect [23,89]. After being distributed in the body, phenolic compounds are known to be extensively metabolized [14,51,84,94,95]. The metabolites produced, such as glucuronides [96], sulfates, aldehydes, acids formed via oxidation of the aliphatic alcohol, methylated forms, acetylated and sulfated derivatives [97], as well as an N-acetylcysteine derivative [98], are found at high concentrations in human tissues. In fact, more than 10 metabolites of hydroxytyrosol and tyrosol have been described. They could act as free forms before entering cells or metabolize once inside them. They seem to reach enough concentrations to exert beneficial effects, through antioxidant properties, as well as modulation of intracellular signaling, improving the cellular response to oxidative stress and pro-inflammatory stimuli [14,99].

Only 5–10% are recovered in urine in their free forms [23]. A clinical trial analysis of urine after olive oil intake identified metabolites of most phenolic compounds, especially hydroxytyrosol, oleuropein aglycone, and oleocanthal. However, low levels of tyrosol, luteolin, apigenin, pinoresinol, and acetoxypinoresinol metabolites were found, suggesting that these compounds may have been excreted through another metabolic pathway or poorly absorbed and excreted in feces [100]. Although in recent years research has also focused on other phenolic compounds [31,100], there is a lack of information about polyphenolic metabolization and the bioavailability of other phenolic compounds has been scarcely studied compared to hydroxytyrosol and tyrosol.

With respect to the latest compounds, the evidence suggests that small amounts of hydroxytyrosol and tyrosol may be synthesized endogenously as products of dopamine and tyramine metabolisms, respectively. This evidence could be confirmed on the basis that even after hours of fasting and after strict diet control, it is possible to find hydroxytyrosol in biological fluids. Consequently, free form concentrations of these polyphenols combine exogenous and endogenous sources [23,95].

Despite the biological benefits already studied associated with phenolic compounds, it is important to develop further research to determine the concentrations that free phenolic compounds reach in the human body and whether it is sufficient to exert biological effects on the target organs, or the possible effect that the resulting metabolites may exert. Aiming to facilitate their biological effects, new strategies have been evaluated to increase the bioavailability of these compounds, thus opening a research area on possible controlled delivery strategies. These strategies have been focused on increasing their absorption through the gastrointestinal tract as well as their transport to the target organs. Such polyphenol delivery systems comprise nanoformulations, namely nanosuspensions, solid lipid nanoparticles, liposomes, gold nanoparticles, and polymeric nanoparticles. These systems are proving to increase the bioactivity of natural polyphenols by increasing their intracellular concentration, thanks to their slow and sustained release [101,102].

### 3.4. Alternative Sources of Olive Biophenols

It is widely known that these compounds are present in olive oil at different quantities depending on cultivar, environmental factors during olive production, and oil processing-related factors as it has been described above. However, phenolic compounds are also present in other parts of the plant or in the by-products resulting from the olive oil elaboration process. These sources of natural and functional substances can potentially be used to recover bioactive compounds [16,103].

The olive oil industry produces a large amount of waste. Wastewater, which is currently a powerful pollutant, could even be an interesting source of phenolic compounds [104,105]. Other sources from the olive tree are its leaves, whose active compounds seem to provide oxidative stability to olive oil, or other edible oils, in addition to its corresponding added nutrition value [106,107]. Phenolic compounds are mainly polar, and consequently only a small amount is solubilized in oil when it is extracted. About 95–96% of phenols present in the olive fruit remain in the residual by-product of olive pomace, being another source of phenolic compounds [16,108]. Olive pomace is produced in large quantities in the olive oil industry, causing an environmental problem. In addition to possible applications as a fertilizer, olive pomace can be used as a natural source of phenolic compounds [103,109,110].

Some researchers focus on the possibility of isolating some of the bioactive compounds from EVOO [82], such as hydroxytyrosol, to enrich the olive oil itself [16] or even to be used as a natural food additive, taking advantage of their antioxidant and antimicrobial activity to improve the stability of processed foods including processed meats [111,112,113,114,115]. In fact, EFSA confirmed in 2017 that hydroxytyrosol can be extracted from olive sources or produced by chemical synthesis or through the use of microorganisms and added in formulas for dietary supplementation with no difference from the natural compound [116].

## 4. Conclusions

Qualitative profile and quantitative amount of individual and total EVOO phenolic compounds vary depending on many factors. Although agronomic, pedoclimatic, and technological conditions to obtain the best olive oil as well as different EVOO phenolic profiles have been described in the literature, future trends to establish a common analytical methodology in order to compare the composition of these bioactive compounds is required to select the best olive tree varieties to obtain a phenol-enriched EVOO.

Studies involving humans, animals, and cell cultures (in vitro and in vivo) have demonstrated that olive phenolic compounds have potentially beneficial effects resulting from their antioxidant activity. Their benefits are closely related to their chemical structure, specifically due to the presence of one or more hydroxyl groups. In addition to the direct scavenging of reactive species, it is clear that the modulation of gene expression plays a key role in the antioxidant and anti-inflammatory properties of olive phenolic compounds. Therefore, accumulating evidence supports the association of phenolic compounds with the prevention or reduced risk of diseases characterized by oxidative stress or inflammation, such as cancer, digestive disorders, metabolic syndrome, and cardiovascular diseases. Notwithstanding, conclusions regarding their preventive potential remain unresolved due to several limitations in existing studies. Further clinical trials are necessary.

In addition to the concentration of phenolic compounds, other factors must be considered when assessing the potential health benefits of dietary EVOO phenols. The bioavailability associated with each phenolic compound, tissue distribution, the effective dose in the target organ, the effect of human genetic variations, differences in gut microbiota that could determine different profile for bioactive phenolic metabolites, synergic effects among phenolic compounds, and the possible interaction between these compounds and other nutrients may alter the receptor function and the possible toxicity associated with its consumption should be also considered. However, there is a lack of clinical data, and then further investigation in this line of research may provide more findings that are conclusive.

Metabolites of phenolic compounds have also been shown to have beneficial biological effects. However, more information is required, since not all compounds are chemically characterized. In this field, controlled delivery strategies of (poly)phenols or even of olive oil itself could be beneficial, enhancing their positive effects and increasing bioavailability.

In conclusion, EVOO intake from Mediterranean diet and even as a functional food, plays critical metabolic roles in the human organism. A large portion of these benefits is associated with its richness in phenolic compounds. Nonetheless, further research is required to establish compound–benefit relationships.

## Figures and Tables

**Table 1:** Classification of phenolic compounds in extra-virgin olive oil (EVOO).

Phenolic Acids		
**Hydroxybenzoic Acid Derivatives**	*p*-Hydroxybenzoic acid (R_1_ = H; R_2_ = H) Protocatechuic acid (R_1_ = OH; R_2_ = H) Vanillic acid (R_1_ = OCH_3_; R_2_ = H) Syringic acid (R_1_ = OCH_3_; R_2_ = OCH_3_) Gallic acid (R_1_ = OH; R_2_ = OH)	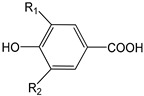
**Hydroxycinnamic Acid Derivatives**	*p*-Coumaric acid (R_1_ = H; R_2_ = H) Ferulic acid (R_1_ = OCH_3_; R_2_ = H) Caffeic acid (R_1_ = OH; R_2_ = H) Sinapic acid (R_1_ = OCH_3_; R_2_ = OCH_3_)	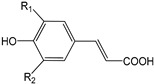
**Lignans**		
	(+)-1-Acetoxypinoresinol (R_1_ = COOCH_3_; R_2_ = H; R_3_ = H) (+)-1-pinoresinol (R_1_ = H; R_2_ = H; R_3_ = H)	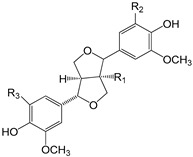
**Flavonoids**		
	Luteolin (R_1_ = OH; R_2_ = H; R_3_ = H) Apigenin (R_1_ = H; R_2_ = H; R_3_ = H)	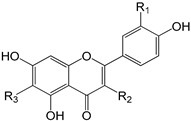
**Phenolic Alcohols**		
	Hydroxytyrosol (R_1_ = OH; R_2_ = H) Tyrosol (R_1_ = H; R_2_ = H)	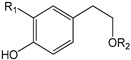
**Secoiridoids**		
	Oleuropein aglycone (R_1_ = OH; R_2_ = COOCH_3_; R_3_ = H) Ligstroside aglycone (R_1_ = H; R_2_ = COOCH_3_; R_3_ = H) Oleacein (R_1_ = OH; R_2_ = H; R_3_ = H) Oleocanthal (R_1_ = H; R_2_ = H; R_3_ = H)	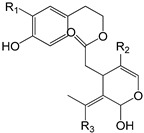
**Hydroxy-Isocromans**		
	1-phenyl-6,7-dihydroxy-isochroman 1-(39-methoxy-49-hydroxy) phenyl-6,7-dihydroxy-isochroman.	
